# Effect of Carbon Nanoparticles on the Crystallization of Calcium Carbonate in Aqueous Solution

**DOI:** 10.3390/nano9020179

**Published:** 2019-02-01

**Authors:** Chuan Wan, Le-Tian Wang, Jun-Yi Sha, Hong-Hua Ge

**Affiliations:** Shanghai Engineering Research Center of Energy-Saving in Heat Exchange Systems, Shanghai Key Laboratory of Materials Protection and Advanced Materials in Electric Power, Shanghai University of Electric Power, Shanghai 200090, China; acvkil@163.com (C.W.); lightWanglt@163.com (L.-T.W.); shashashajunyi@163.com (J.-Y.S.)

**Keywords:** carbon nanoparticles, nanofluid, scale inhibition, calcium carbonate, crystallization

## Abstract

Nanofluids have great application prospects in industrial heat exchange systems because they can significantly improve the heat and mass transfer efficiency. However, the presence of nanoparticles in the fluid might also affect the formation and attachment of inorganic scales, such as calcium carbonate, on the heat exchange surface. The effects of carbon nanoparticles on the crystallization of calcium carbonate in aqueous solution were studied by the scale inhibition test, solution analysis, scanning electron microscopy (SEM), X-ray photoelectron spectroscopy (XPS), and Fourier transform infrared spectroscopy (FTIR). The results showed that carbon nanoparticles had an excellent surface scale inhibition performance for calcium carbonate, which could effectively prevent the adhesion of scale on the heat exchange surface. The carbon nanoparticles did not affect the solubility of calcium carbonate in water, but changed the crystal form of the precipitated calcium carbonate, making it difficult to adsorb on the heat exchange surface and achieving a surface scale inhibition effect. Carbon nanofluids effectively inhibit the adhesion of calcium carbonate to heat exchange surfaces.

## 1. Introduction

Today, the world is facing an increasingly serious water shortage problem, and water recycling is considered an effective way to alleviate this problem [1,2]. However, the application of water recycling in the industrial field, such as the circulated cooling water system, is bound to increase the concentration of ions in the water. As the ion concentration increases, the scale-forming ions, such as calcium ions and carbonate ions, in the water easily combine and precipitate as calcium carbonate crystals and adhere to the heat exchange surface to form a scale layer, which not only causes an economic loss due to a decrease in heat transfer efficiency, but might also cause serious production problems due to pipeline blockage [3,4,5,6]. Current scale inhibition methods can be divided into the following two types: physical scale inhibition and chemical scale inhibition. The physical scale inhibition method mainly achieves the scale inhibition effect by the physical action of some energy fields (magnetic fields, electric fields) on the scale-forming substances. For example, the association degree of water molecules and the surface tension of the aqueous solution will be changed under the electric field and these changes will influence the formation process of the inorganic scale. Physical scale inhibition is a promising environmentally friendly scale inhibition technique. However, the physical scale inhibition method is not very effective and its scale inhibition mechanism is still unclear; therefore, it is not widely used at present [7,8,9]. The chemical scale inhibition method is currently the most commonly used method. It mainly adds a certain amount of scale inhibitor to the water system. The scale inhibition effect is achieved by the interaction between the active groups in the scale inhibitor and calcium ions in the water. It can achieve a good scale inhibition performance at an extremely low dosage (mg/L) of the scale inhibitor. At present, mature commercial scale inhibitors are mainly organic phosphonate, which brings unavoidable environmental pollution during use. With the improvement of environmental protection requirements, the development of new environmentally-friendly green scale inhibitors has become a hot spot in the current research of scale inhibitors [10,11,12,13,14].

The concept of a nanofluid was proposed by Choi et al. in 1997. It refers to a novel fluid obtained by dispersing nanoparticles in a base liquid [15], which exhibits excellent thermal conductivity compared to conventional fluids [16,17,18,19,20]. In the past two decades, many researchers have carried out research on the application of nanofluids in the field of heat transfer. Nanofluids have applications in the fields of industrial heat exchange [21], solar energy [22], automotive engines [23], and the cooling of electronic equipment [24]. One major problem in the practical application of nanofluids is the stability of nanoparticles, which directly affects the thermal conductivity and other properties of nanofluids [25]. Ultrasonic and suitable surfactants are effective methods for dispersing nanoparticles [26,27].

Calcium carbonate is the most important fouling substance in cooling water systems. The addition of nanoparticles into a scale-forming solution may result in changes in the crystallization process of calcium carbonate, which may alter the adhesion process of the calcium carbonate scale on the heat transfer surface. It has been reported that nanoparticles or nanofluids were utilized to affect the crystallization process of calcium carbonate. In the biomedical field, chitosan nanoparticles were used as the substrate of calcium carbonate crystallization and it was found that chitosan nanoparticles can change the crystal form of calcium carbonate from calcite to vaterite [28]. Carbon nanotubes (CNTs) were used as carriers to adsorb the polyphosphinocarboxylic acid scale inhibitor and they exhibited better scale inhibition performance [29]. The strong adsorption of CNTs caused the scale inhibitor to have longer retention and a higher adsorption concentration. A nanostructured scale inhibitor, Ca-DTPMP, based on diethylenetriamine penta(methylenephosphonate) (DTPMP), was prepared by Kiaei et al. [30] and its effect on the growth of calcium carbonate in bulk solution was studied. It was found that Ca-DTPMP nano-inhibitors can increase the concentration of residual Ca^2+^ in the scale forming solution and interfere with the formation of calcite crystals. The effect of SiO_2_ nanoparticles on the crystallization process of CaCO_3_ was also explored [31]. The results showed that the addition of SiO_2_ nanoparticles shortened the induction period of CaCO_3_ crystallization and enhanced the control of the crystallization process. This may result in preferential precipitation at a faster rate of CaCO_3_ on the surface of the nanoparticles, thereby reducing the deposition of scale on the tube wall and achieving scale inhibition. Researchers have also reported that the increase in the calcium ion concentration in the solution will have a greater impact on the experimental results. Surface-activated multiwall carbon nanotubes (MWCNTs) can serve as templates for calcium silicate growth [32] and the addition of MWCNTs could control the morphology of Ca_2_SiO_4_ formed by CaCO_3_ and silicic acid. Some carbon nanomaterials, such as modified water-soluble carbon nanomaterials, hydroxylated single-walled carbon nanotubes (HO-SWNTs), and isophthalic acid functionalized-SWNTs, could alter the morphology of the deposited CaCO_3_ crystals [33], but the purified single-walled carbon nanotubes (SWNTs) could not exert an effect on both the phase and morphology of CaCO_3_. Amorphous carbon nanoparticles are inexpensive and have a wide range of sources. The effect of amorphous carbon nanoparticles or carbon nanofluids on the precipitation and adhesion behaviour of scale-forming substances has not been reported. In this paper, the effects of a type of carbon nanoparticles on the crystallization of calcium carbonate in a scale-forming solution were investigated. The feasibility of using carbon nanoparticles for surface scale inhibition was discussed.

## 2. Materials and Methods

### 2.1. Experimental Materials

The experimental carbon nanoparticles were about 20 nm in size and were amorphous materials obtained from coconut shells. The dispersant used was polyvinylpyrrolidone (PVP). Both were provided by the Suzhou Tanfeng Technology Company (Suzhou, China). Calcium chloride and the remaining chemicals used in the experiment were analytical reagents (AR) supplied by Sinopharm Chemical Reagent Co., Ltd. (Shanghai, China) Deionized water was used to prepare all test solutions.

### 2.2. Particle Size Characterisation of Carbon Nanoparticles

A small amount of carbon nanoparticles was dispersed in an alcohol solution and ultrasonically dispersed. The sample was observed under a transmission electron microscope (TEM) (JEM-2100F, JEOL, Tokyo, Japan) to ensure the particle size of the nanoparticles.

### 2.3. The Preparation of Carbon Nanofluids

According to Yoon et al. [34], PVP was selected as a dispersant for dispersing carbon nanoparticles. The weighed carbon nanoparticles were placed in an agate mortar and ground evenly. Then, a 1 mL solution (at a concentration of 0.25 g/L) was added to disperse them. This was transferred to a glass beaker and filled to 200 mL with deionized water and agitated at a speed of 500 rpm/min for 5 min, followed by ultrasonic dispersion for 10 min. It was then set aside.

### 2.4. The Scale Inhibition Experiment of CaCO_3_

According to the Chinese National Standard (the determination of scale inhibition performance of water treatment agents—Calcium carbonate precipitation method, GB/T 16632-2008), the scale-forming solution was prepared and controlled at 50 °C during the experiment. The initial pH of the scale-forming solution containing 6 mmol/L CaCl_2_ and 12 mmol/L NaHCO_3_ was maintained at 9.0 by 10 mmol/L Na_2_B_4_O_7_·10H_2_O. The prepared scale-forming solution was placed in 304 stainless steel cups of known quality and the experimental 304 stainless steel cups were placed in a water bath at a constant temperature of 50 °C for 12 h. Then the stainless steel cups were removed from the water bath and the test solution was poured out. The cup walls were rinsed with deionized water and then dried and weighed. The increased mass of the 304 stainless steel cups after the experiment was the amount of calcium carbonate scale attached to the cup.

Surface scale inhibition efficiency is calculated as follows:

(1) $$\eta =\frac{\Delta m_{1}-\Delta m_{2}}{\Delta m_{1}}\times 100\%,$$

where $\Delta m_{1}$ (g) is the increased weight of the stainless steel cup in the blank experiment, and $\Delta m_{2}$ (g) is the increased weight of the stainless steel cup in the scale-forming solution containing carbon nanoparticles.

### 2.5. Solution Analysis

#### 2.5.1. Determination of Residual Calcium Concentration in Solution

The test solutions were filtered after the experiments. The calcium carboxylic acid indicator was added to each filtrate. The content of calcium ion in the filtrate was titrated and determined by a 0.01 mol/L ethylene diaminetetraacetic acid disodium salt (EDTA) standard solution according to GB/T 16632-2008.

#### 2.5.2. Determination of Critical Point of Calcium Carbonate Crystallization

The 6 mmol/L CaCl_2_ solution was placed in a constant temperature magnetic agitator and the water bath was heated and maintained a constant temperature of 50 °C. The 0.1 mol/L Na_2_CO_3_ solution was added dropwise to the CaCl_2_ solution while stirring at 500 rpm/min. The conductivity of the solution was measured after each addition of 0.2 mL Na_2_CO_3_ solution. The Na_2_CO_3_ solution was continuously added until a sudden decrease in the conductivity occurred. The point corresponding to the first decrease in the conductivity is the critical point for the crystallization of calcium carbonate [35]. The volume of the Na_2_CO_3_ solution consumed before this point was recorded.

### 2.6. Morphology and Structure Analysis of Calcium Carbonate Crystals

In a 50 °C water bath, the 36 mmol/L NaHCO_3_ solution was slowly added dropwise to the 18 mmol/L CaCl_2_ solution with or without nanoparticles, and the calcium carbonate crystals were gradually precipitated in the solution, then filtered and dried to obtain the test scale sample.

The surface morphology of the scale samples under different conditions was observed by a scanning electron microscope (SEM) (JSM-7800F, JEOL, Tokyo, Japan). The crystal structure of the scale samples was analysed by Fourier transform infrared (FTIR) spectroscopy (Spectrum Two, PerkinElmer, Waltham, MA, USA) using KBr pellets as a blank sample. The crystal types of the scale were analysed by X-ray diffraction (XRD) (D 8 ADVANCE, Bruker, Billerica, MA, USA).

## 3. Results and Discussion

### 3.1. The Stability of Carbon Nanoparticles in Scale-Forming Solution

**Figure 1:**
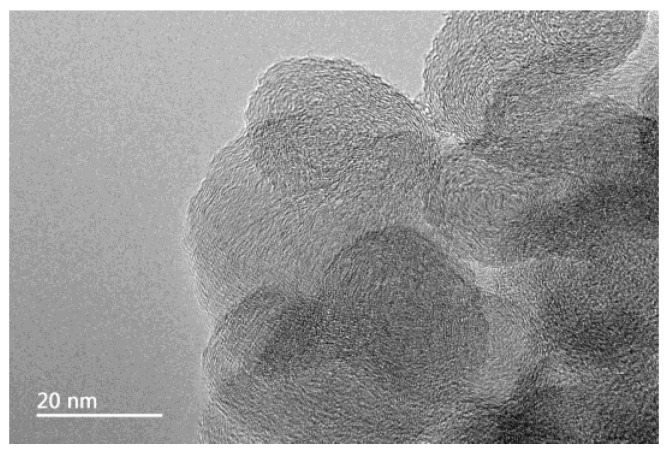
The transmission electron microscope (TEM) image of carbon nanoparticles.

**Figure 2:**
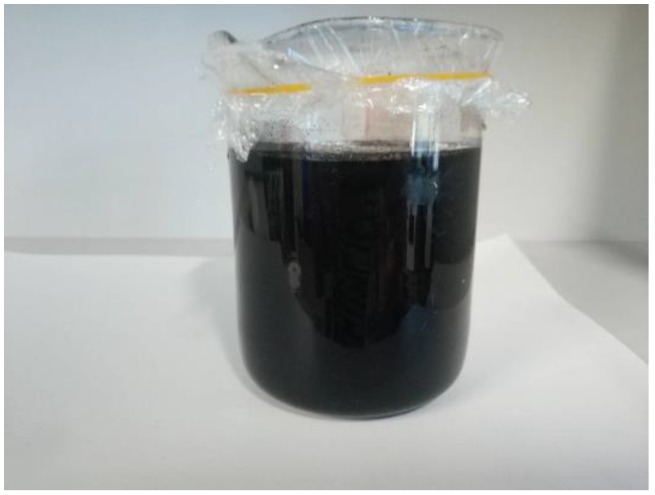
The dispersed carbon nanoparticles in the scale-forming solution.

The TEM image of carbon nanoparticles is shown in Figure 1. The particle size of carbon nanoparticles is mostly around 20 nm. Figure 2 shows the dispersion solution of carbon nanoparticles in the scale-forming solution. The carbon nanoparticles were stably dispersed in the solution without sedimentation.

### 3.2. The Scale Inhibition Performance of Carbon Nanoparticles

First, the effect of dispersion conditions, such as the addition of dispersant PVP, ultrasonic treatment and joint treatment of PVP and ultrasonic, on the scale inhibition performance of calcium carbonate was determined and the results are given in Table 1. It was found that the amount of scale attached to the surface of the experimental cups under these three dispersion conditions was almost the same as the blank result. The use of dispersion conditions produced almost no scale inhibition effect; therefore, in the subsequent experiments, the effect of dispersion conditions on scale inhibition performance could be ignored. 

**Figure 3:**
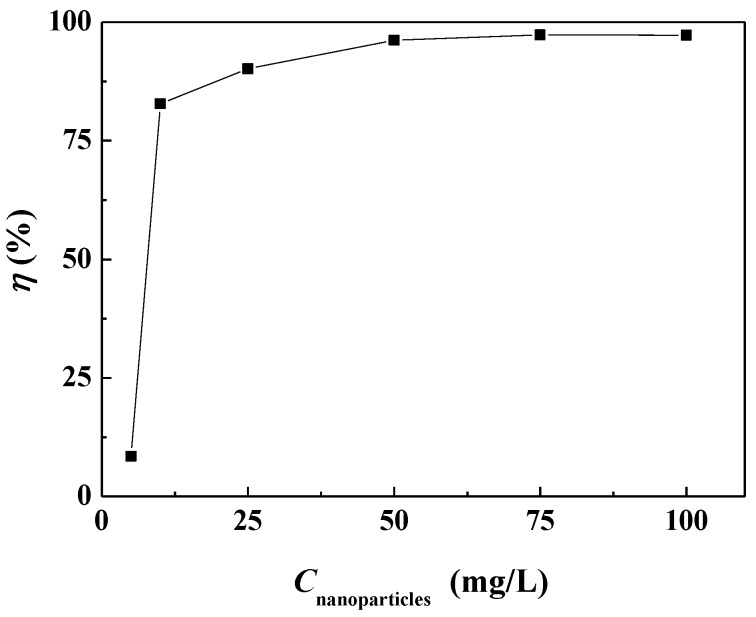
The surface scale inhibition efficiency of carbon nanoparticles at different concentrations.

**Figure 4:**
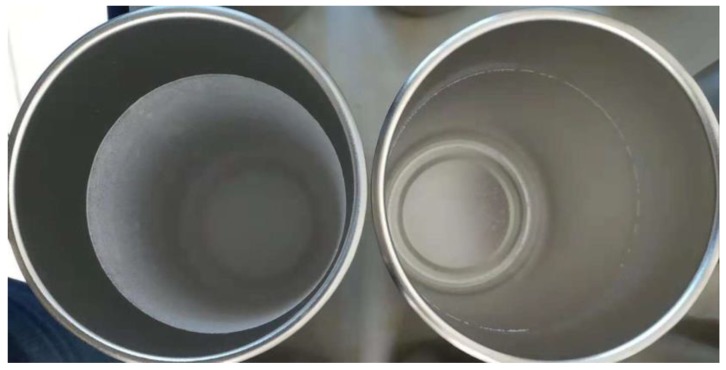
The image of the CaCO_3_ scales attached to the experimental cup in the absence of an inhibitor (left) and in the presence of 100 mg/L carbon nanoparticles (right).

The surface scale inhibition performance of carbon nanoparticles at different concentrations on calcium carbonate was investigated. Figure 3 shows that the surface scale inhibition efficiency of carbon nanoparticles varied with the concentration of nanoparticles in the scale-forming solution. When the concentration of carbon nanoparticles was 5 mg/L, the scale inhibition efficiency was about 8.50%, which means a poor scale inhibition performance. The scale inhibition efficiency increased to 82.82% when the nanoparticle concentration was 10 mg/L. With the continuous addition of carbon nanoparticles, the scale inhibition efficiency increased gradually. When the nanoparticle concentration reached 50 mg/L, the scale inhibition efficiency surpassed 95%. When the concentration was 75 mg/L, the carbon nanoparticles exhibited the best scale inhibition efficiency of 97.31%. Figure 4 visually demonstrates the scale inhibition effect of carbon nanoparticles. In this scale-forming solution, 50 mg/L is the ideal nanoparticle concentration for scale inhibition.

In fact, a certain concentration of carbon nanoparticles would exhibit different scale inhibition efficiencies when the scale-forming ion concentrations change. There was no obvious change in the scale inhibition efficiency in the scale-forming solution at lower concentrations. Experimental results using 2 times concentrated scale-forming solution showed that the scale inhibition efficiency of 50 mg/L carbon nanoparticles was reduced to 81.3%, while the scale inhibition efficiency of 100 mg/L carbon nanoparticles was maintained at 97%. So, in practical applications, it is necessary to determine the required concentration of carbon nanoparticles based on the concentration of scale-forming ions in the water.

### 3.3. Effect of Carbon Nanoparticles on the Concentration of Ca^2+^ in Solution

In general, chemical scale inhibitors cannot prevent the formation of scaling substances, but delay the crystallization period of scale ions or alter the growth mechanism of crystals [36]. Because of the strong complexing effect of their functional groups with calcium ions, the solubility of calcium ions in water can increase, which is considered to be the main reason for the scale inhibition of chemical scale inhibitors. In addition, the scale inhibitors can also adsorb on the crystal surface of the sediment, resulting in the change in the crystal form of calcium carbonate [37]. Therefore, most of the chemical scale inhibitors have both solution scale inhibition performance and surface scale inhibition performance. To investigate the scale inhibition mechanism of carbon nanoparticles, the residual calcium ion concentration in the test solution was determined and the influence of carbon nanoparticles on the critical point of calcium carbonate crystallization was analysed by conductivity experiments.

#### 3.3.1. Effect of Carbon Nanoparticles on Residual Calcium Ion in Solution

**Figure 5:**
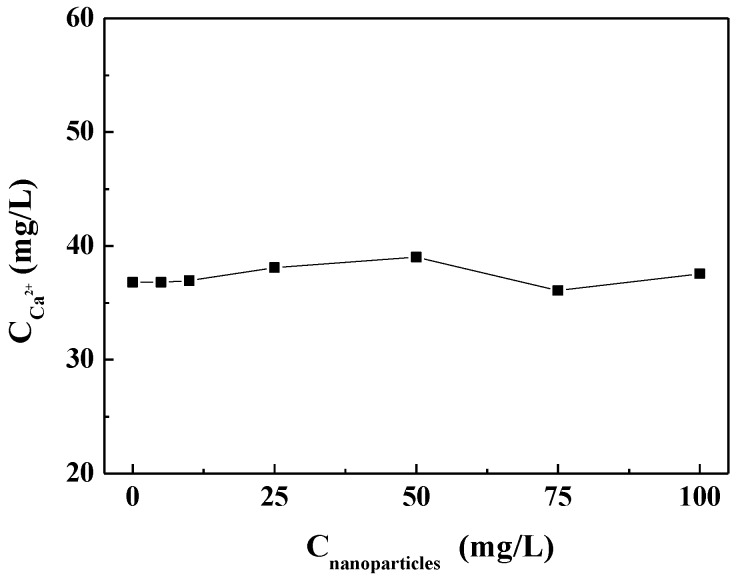
The concentration of residual calcium ions after experiments in the scale-forming solution containing different concentrations of carbon nanoparticles.

Figure 5 shows the concentration of residual calcium ions after the experiment in the scale-forming solution containing different concentrations of carbon nanoparticles. It can be seen that the concentration of residual calcium ions in the test solutions did not change substantially with the carbon nanoparticle concentrations. That is, the addition of the carbon nanoparticles did not change the calcium ion concentration in the scale-forming solution and, therefore, did not exhibit solution scale inhibition performance.

#### 3.3.2. Effect of Carbon Nanoparticles on Critical Point of Calcium Carbonate Crystallization

The critical point of calcium carbonate crystallization was determined by the conductivity method. During the measurements, the sodium carbonate solution was gradually added to the calcium chloride solution, and the conductivity of the solution gradually increased with the addition of sodium carbonate. When the concentration of calcium carbonate in the solution reached a certain degree of supersaturation, the conductivity of the solution reached a maximum. With the continued addition of sodium carbonate solution, the nucleation and crystallization of calcium carbonate occurred and the amount of freely ionisable calcium and carbonate ions in the solution decreased, causing the conductivity of the solution to drop sharply. The critical point of calcium carbonate crystallization can be determined by the abrupt drop point of the conductivity, which is called the critical conductivity value [36].

**Figure 6:**
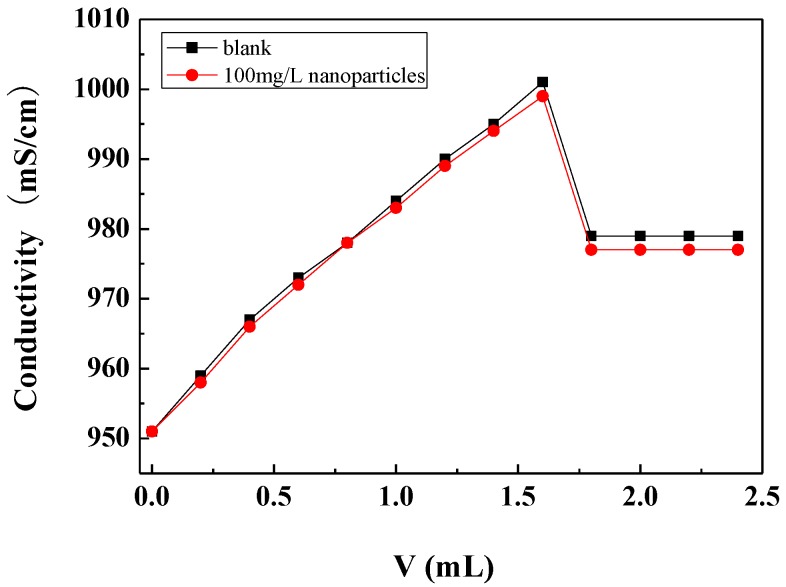
The conductivity changes with the addition amount of Na_2_CO_3_ solution in the scale-forming solution.

Figure 6 shows the conductivity changes with the addition of the Na_2_CO_3_ solution in the scale-forming solution containing carbon nanoparticles and in the blank. It can be seen that the critical conductivity value of the scale-forming solution did not change obviously after the addition of the carbon nanoparticles. Therefore, the addition of carbon nanoparticles hardly changed the critical point of calcium carbonate crystallization, that is, carbon nanoparticles did not change the supersaturation of calcium carbonate in solution. The results are consistent with the residual calcium ion concentration measurement results shown in Section 3.3.1.

### 3.4. Morphology and Structure Analysis of Calcium Carbonate Crystals

According to the above results, the carbon nanoparticles in the scale-forming solution had neither the ability to increase the residual calcium ion concentration nor the function of accelerating the nucleation of the scale-forming particles (calcium carbonate); therefore the scale inhibition effect of carbon nanoparticles may come from the changes in the morphology and crystal form of calcium carbonate, as described in the work of Anderson [33].

There are three crystal forms of calcium carbonate precipitated in aqueous solution: calcite, aragonite, and vaterite. Among them, aragonite and vaterite are metastable structures and calcite is the most stable type in thermodynamics. In the absence of external factors, other crystal forms will be transformed into calcite [38,39].

#### 3.4.1. SEM Observation

**Figure 7:**
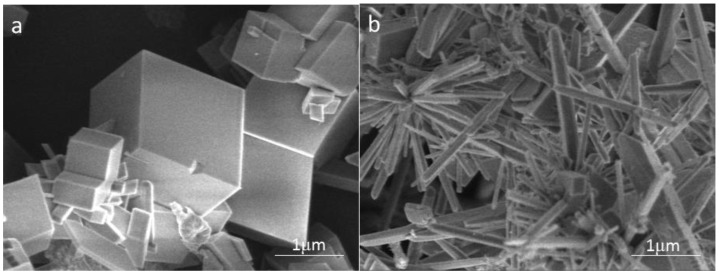
The scanning electron microscope (SEM) images of the calcium carbonate crystals formed in the blank (**a**) and the scale-forming solution containing 100 mg/L carbon nanoparticles (**b**).

To investigate the effect of carbon nanoparticles on the crystal form of calcium carbonate in solution, the calcium carbonate crystals formed in the blank and the scale-forming solution containing 100 mg/L carbon nanoparticles were characterised by SEM. The results are shown in Figure 7. The shape of calcium carbonate crystals produced in the blank solution is mostly rhombohedral (Figure 7a); the surface is smooth with sharp edges and corners, showing the crystal form of calcite. Figure 7b displays the calcium carbonate formed in the solution containing 100 mg/L carbon nanoparticles. Most of these scale samples are rod-shaped and a tiny amount exhibits rhombohedral structures. It can be seen that the addition of carbon nanoparticles caused a significant change in the crystal form of calcium carbonate. Anderson et al. [33] reported that hydroxylated single-walled carbon nanotubes promote the formation of amorphous calcium carbonate and reduce the adsorption of calcium carbonate on a solid surface. The change in the morphology caused by carbon nanoparticles should also affect the adhesion behaviour of calcium carbonate on the metal surface.

#### 3.4.2. FTIR Analysis

**Figure 8:**
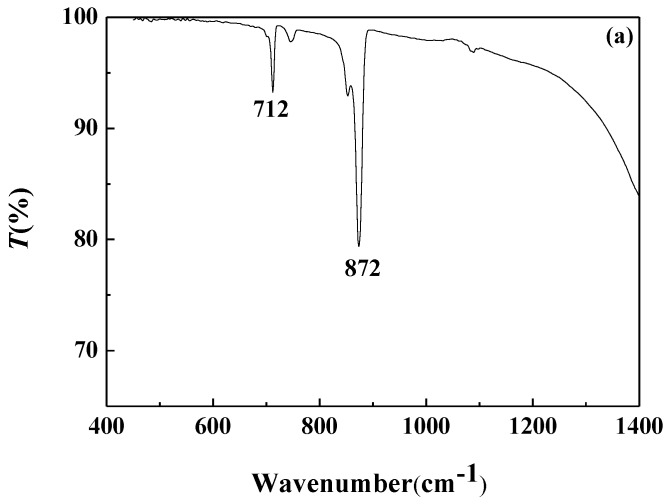

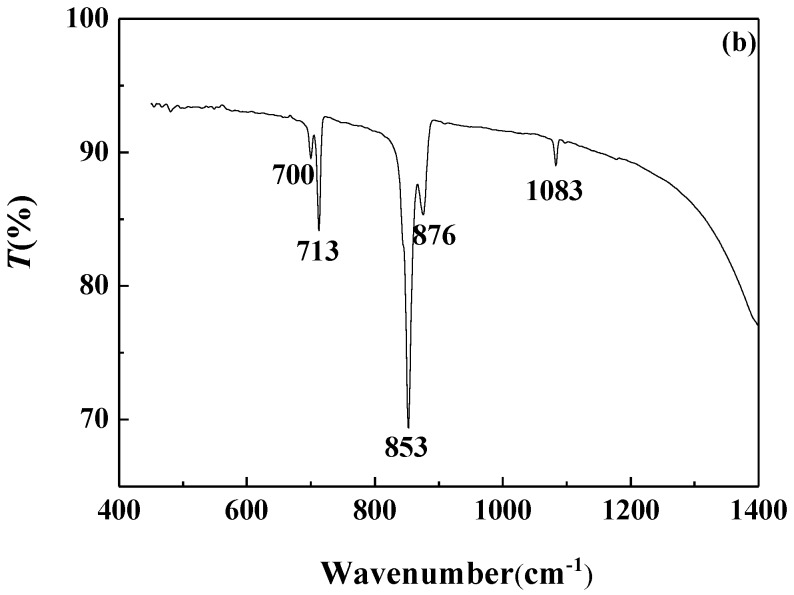
The Fourier transform infrared (FTIR) spectra of the calcium carbonate crystals formed in the blank (**a**) and the scale-forming solution containing 100 mg/L carbon nanoparticles (**b**).

FTIR analysis of the calcium carbonate crystals prepared in the blank scale-forming solution and the scale-forming solution containing 100 mg/L carbon nanoparticles were carried out and the results are shown in Figure 8. The calcium carbonate crystals formed in the blank had the characteristic absorption peak of calcite at wavelengths of 712 cm^−1^ and 872 cm^−1^, which corresponds to the deformation vibration of the O–C–O surface and the deformation vibration of the ${CO}_{3}^{2-}$ surface, respectively [40]. For the calcium carbonate crystals formed in the solution containing carbon nanoparticles, the absorption peaks of calcite at wavelengths of 712 cm^−1^ and 872 cm^−1^ almost disappeared, and the characteristic absorption peaks of aragonites at 700 cm^−1^, 713 cm^−1^, and 853 cm^−1^, as well as a small absorption peak of vaterite at 1083 cm^−1^, were seen [41].

#### 3.4.3. XRD Analysis

**Figure 9:**
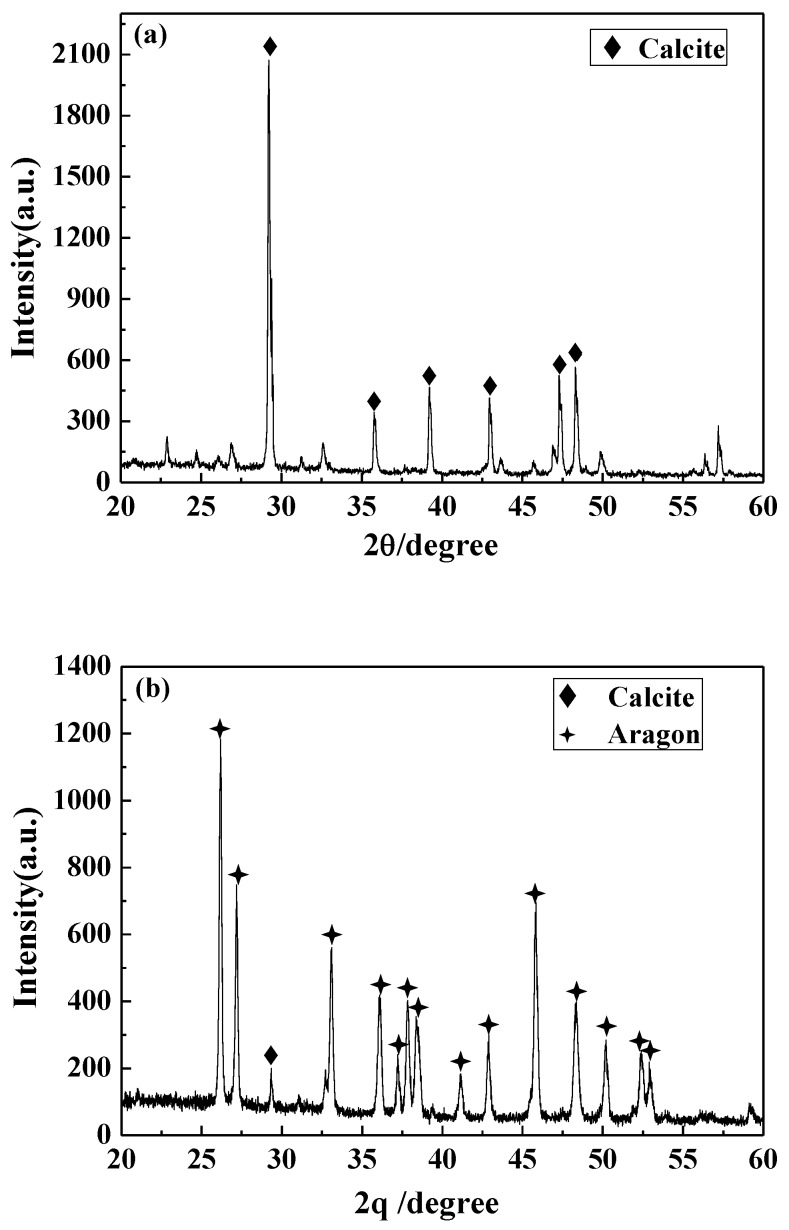
The X-ray diffraction (XRD) pattern of the calcium carbonate crystals formed in the blank (**a**) and the scale-forming solution containing 100 mg/L carbon nanoparticles (**b**).

The structural changes of the precipitated calcium carbonate crystals in different solutions were detected by XRD. The results are displayed in Figure 9. The calcium carbonate crystals in the blank have a strong diffraction peak at 29.4°, which corresponds to the 104 crystal plane of calcite. In addition, there are peaks at 35.8° (110 crystal plane of calcite), 39.3° (113 crystal plane of calcite), 43.0° (202 crystal plane of calcite), 47.4° (108 crystal face of calcite), and 48.5° (116 crystal face of calcite). It was indicated that the calcium carbonate formed in the blank is calcite, and the 104 crystal plane is its main growth surface. For the calcium carbonate crystals formed in the solution containing carbon nanoparticles, most of the diffraction peaks of calcite disappeared and the characteristic peaks of aragonite appeared at 26.1°, 27.1°, 33.0°, 36.0°, 37.8°, 38.5°, 42.8°, 45.7°, 48.3°, 50.1°, 52.3°, and 52.9° corresponding to the (111), (021), (012), (200), (031), (112), (220), (221), (202), (132), and (231) crystal faces of aragonite [32,41,42]. This indicates that the presence of carbon nanoparticles transformed the main crystal form of calcium carbonate from calcite to aragonite, which is consistent with the SEM and FTIR results.

#### 3.4.4. Effect of Carbon Nanoparticles on the Crystallization Process of Calcium Carbonate

The precipitation of calcium carbonate is a four-stage crystallization process [39,43]. (1) First is aggregation. When the super-saturation of the solution reaches a certain degree, the Ca^2+^ and ${CO}_{3}^{2-}$ ions in the solution aggregate together to form an ion-pair solution. Then, these pairs continue to form microcrystalline cores. (2) Next is nucleation. Some microaggregates continue to be nucleation centres of crystallization, which lead to the formation of microcrystals of amorphous calcium carbonate. Unstable amorphous calcium carbonate is quickly converted to polycrystalline calcium carbonate. (3) The third stage is crystal growth. Polycrystalline calcium carbonate formed in the solution aggregates or absorbs to the solid surface to grow into larger crystallites and finally merges large crystals on the solid surface. (4) The final stage is coagulation. These macroscopic crystals continue to grow by adsorbing additional scaling ions from the solution and eventually forming a scale layer on the surface. This stage is mainly the growth, aggregation, and deposition stage of calcite. A small amount of amorphous calcium carbonate can also be directly converted into calcite.

The calcite has a stable structure. Its crystal shape is regular and easy to pile up. The resulting scale layer formed on the solid surface is dense and hard and can be firmly attached to the heat exchange surface. The crystals of aragonite and vaterite are irregular in shape and loose in structure, and the generated scale is difficult to adhere closely to the heat exchange surface. Therefore, controlling calcium carbonate in the amorphous stage or polycrystalline calcium carbonate in the form of aragonite or vaterite can retard or prevent the adhesion of calcium carbonate crystals on the surface, thereby achieving the effect of surface scale inhibition. The carbon nanoparticles can change the morphology and structure of the calcium carbonate crystals, make aragonite and vaterite be stably present in solution and on the metal surface, thereby exerting a surface scale inhibition effect.

## 4. Conclusions

(1) The carbon nanoparticles in the scale forming solution can inhibit the formation of calcium carbonate scale on the heat exchange surface and have a good surface scale inhibition effect. When the concentration of carbon nanoparticles is 75 mg/L, the scale inhibition efficiency can reach 97.31%.

(2) Unlike the chemical scale inhibitors, the carbon particles have little effect on the solubility of calcium carbonate in the scale-forming solution. The residual calcium ion concentration of the blank solution and the solution containing carbon nanoparticles is almost the same after the experiments. The critical conductivity is also unaffected by the carbon nanoparticles.

(3) The surface scale inhibition effect of carbon nanoparticles is mainly due to its ability to change the crystal form of calcium carbonate. The calcium carbonate crystals formed in the blank scale forming solution are mainly calcite. The presence of the carbon nanoparticles changes the crystal of calcium carbonate from calcite to aragonite, making it difficult to adhere to the heat exchange surface.

## Figures and Tables

**Table 1:** The surface scale inhibition efficiency in different dispersion conditions.

Dispersion Conditions	PVP	Ultrasonic	Ultrasonic + PVP
Surface scale inhibition efficiency (η)	0.12%	−0.33%	0.46%
